# Prognostic Values of Inflammation-Based Scores and Fibrosis Markers in Patients with Hepatocellular Carcinoma Treated with Transarterial Chemoembolization

**DOI:** 10.3390/diagnostics12051170

**Published:** 2022-05-07

**Authors:** Eun Ju Cho, Su Jong Yu, Yun Bin Lee, Jeong-Hoon Lee, Yoon Jun Kim, Jung-Hwan Yoon

**Affiliations:** Department of Internal Medicine and Liver Research Institute, Seoul National University College of Medicine, Seoul 03080, Korea; creatio3@snu.ac.kr (E.J.C.); yblee@snu.ac.kr (Y.B.L.); jhleemd@snu.ac.kr (J.-H.L.); yoonjun@snu.ac.kr (Y.J.K.); yoonjh@snu.ac.kr (J.-H.Y.)

**Keywords:** hepatocellular carcinoma, transarterial chemoembolization, neutrophil–lymphocyte ratio, FIB-4 index

## Abstract

Background: Inflammation is a key feature shaping the microenvironment of hepatocellular carcinoma (HCC), and liver fibrosis is associated with the prognosis of patients with HCC. In this study, we investigated whether baseline inflammation-based scores and serum fibrosis markers can help in predicting the prognosis of HCC patients treated with transarterial chemoembolization (TACE). Methods: A total of 605 consecutive patients with HCC treated by TACE were included. The systemic immune-inflammation index (SII), neutrophil–lymphocyte ratio (NLR), platelet–lymphocyte ratio (PLR), FIB-4 index, and aspartate aminotransferase-to-platelet ratio index (APRI) were analyzed regarding their associations with disease progression and survival. Results: All tested inflammation-based scores and fibrosis markers were significantly associated with tumor progression and overall survival in the univariate analyses. In the multivariate analysis, NLR (hazard ratio [HR], 1.06; *p* = 0.007) and FIB-4 (HR = 1.02, *p* = 0.008) were independent risk factors for disease progression, along with α-fetoprotein (AFP) levels, maximum tumor size and number, and presence of vascular invasion. Furthermore, NLR (HR, 1.09; *p* < 0.001) and FIB-4 (HR, 1.02; *p* = 0.02) were independent prognostic factors for survival. Conclusions: High baseline NLR and FIB-4 levels might help the prediction of disease progression and death in patients with HCC after TACE.

## 1. Introduction

Inflammation is a principal feature of chronic liver disease and predisposition to hepatocellular carcinoma (HCC) [1]. An ineffective immune response during hepatitis viral infection or alcohol consumption leads to chronic inflammation through hepatocyte apoptosis. The increase in regulatory T cells in the inflammatory hepatic milieu is associated with inhibiting the resolution of fibrosis and the progression of liver disease, leading to immune dysfunction and hepatocarcinogenesis in HCC [2,3]. Therefore, various inflammation-based scores have been suggested as potential prognostic factors to predict the recurrence and survival of patients with HCC, such as the neutrophil–lymphocyte ratio (NLR), platelet–lymphocyte ratio (PLR), systemic immune-inflammation index (SII), and prognostic nutritional index [4,5]. Although these scores are nonspecific markers of inflammation, their components are routinely measured in daily practice. Therefore, these readily available scores could be highly applicable to the risk stratification of patients with HCC.

Chronic inflammation-induced hepatic fibrosis is a well-known host factor contributing to hepatocarcinogenesis. Increased extracellular matrix stiffness promotes hepatic stellate cell activation and hepatocyte proliferation and reduces tumor surveillance by natural killer cells, leading to a tumor-promoting microenvironment [6]. Because fibrosis contributes to the occurrence and recurrence of HCC, assessment of the degree of fibrosis is important for the prognostication of patients. Various noninvasive fibrosis markers including the aspartate aminotransferase-to-platelet ratio index (APRI), FIB-4 index, and Forns index have been used to assess the severity of fibrosis and to predict HCC recurrence and the survival of patients, especially for those who underwent resection or radiofrequency ablation (RFA) [7,8].

Therefore, the combination of inflammation-based prognostic scores and fibrosis markers may improve the prognostication of patients by simultaneously considering two major components associated with hepatocarcinogenesis. However, few studies have evaluated the integrated prognostic value of inflammation-based scores and noninvasive fibrosis markers in patients who have undergone transarterial chemoembolization (TACE) for HCC. Thus, we investigated whether baseline inflammation-based scores and serum fibrosis markers can be used to predict the disease progression and survival of patients with HCC following TACE.

## 2. Materials and Methods

### 2.1. Study Population

This retrospective study included 605 patients with HCC who had undergone TACE as their initial treatment between January 2012 and December 2013, at Seoul National University Hospital (Seoul, Korea). HCC was diagnosed according to the noninvasive criteria of the American Association for the Study of Liver Diseases [9]. Cirrhosis was diagnosed either clinically (evidence of hepatic decompensation, radiologic findings of a nodular liver and/or features of portal hypertension, or endoscopic findings of varices), or histologically. The indication for TACE included mainly non-resectable patients with large or multiple tumors with well-preserved liver function, but patients with very early or early stage HCC who would not be candidates for a resection or local ablation because of a comorbidity, poor liver function, or technical difficulty, also underwent TACE. Treatment with TACE was not considered in patients with extensive multifocal HCC affecting both lobes, with severe ascites or jaundice, who were expected to have a deteriorating liver function after treatment. Exclusion criteria were as follows: (1) a history of hematological disorder, autoimmune diseases, or human immunodeficiency virus infection; (2) patients with pyrexia, infection, or gastrointestinal hemorrhage at the time of admission; (3) patients who received antibiotics or anti-inflammatory therapy within the 2 weeks before TACE; and (4) those lost to follow-up after the first session.

Laboratory examinations were routinely performed a day before treatment to check the hematological and biochemical profiles. NLR, PLR, SII, APRI, and FIB-4 were calculated as previously published [5,10].

### 2.2. TACE Procedure

Details regarding the TACE procedure have been described previously [11]. Briefly, conventional TACE was selectively performed by infusion of a mixture of 10–60 mg of doxorubicin emulsion and 2–12 mL of iodized oil contrast medium (Lipiodol; Guerbet LLC, Bloomington, IN, USA) through a microcatheter (Microferret; Cook, Bloomington, IN, or Progreat; Terumo, Tokyo, Japan), followed by embolization using absorbable gelatin sponge particles 1 mm in diameter (Gelfoam; Upjohn, Kalamazoo, MI, or Cutanplast; Mascia Brunelli, Milano, Italy) soaked in a mixture of 10–20 mg doxorubicin hydrochloride and 10 mL of nonionic contrast medium.

Contrast-enhanced computed tomography (CT) was performed 4–8 weeks after TACE to assess the need for subsequent treatment. Repeated TACE was performed every 8–12 weeks if viable HCC (i.e., contrast enhancement during the arterial phase, washout in the portal, and delayed phase) was detected on imaging without liver function deterioration.

### 2.3. Statistical Analyses

The primary outcome was time to progression (TTP), defined as the time from the date of TACE until the first documented tumor progression on imaging studies, according to the modified response evaluation criteria in solid tumors [12]. The secondary outcome was overall survival (OS), measured from the date of TACE to the date of death. The Mann–Whitney U test and Kruskal–Wallis test were used to analyze the differences between the groups. The χ2 test or Fisher’s exact test was used for categorical data. The cumulative rate of survival was calculated using the Kaplan–Meier method, and the log-rank test was performed to compare differences between the groups. Cox proportional hazards models were used to assess the influence of the clinical variables on outcomes. Time-dependent receiver-operating characteristic (ROC) curves were constructed to define the best cut-off value for predicting the outcome [13]. Differences at *p* < 0.05 were considered significant. The statistical analyses were performed using SPSS for Windows, version 23.0 (SPSS Inc., Chicago, IL, USA).

## 3. Results

### 3.1. Baseline Characteristics and Outcomes

The patients’ median age at diagnosis was 57 years (interquartile range [IQR], 50–64 years), and the majority (82.1%) were male (Table 1). As expected, hepatitis B virus (HBV) was the most common etiology regarding underlying liver diseases (72.6%), and the median MELD-Na score was 10. According to the Barcelona Clinic liver cancer (BCLC) staging system, 7.1%, 29.6%, 15.2%, and 48.1% of the patients belonged to stage 0, A, B, and C, respectively. The associations between the levels of systemic inflammation-based scores, noninvasive fibrosis markers, and clinical characteristics are shown in Supplementary Table S1. The levels of APRI and FIB-4 were significantly higher in patients with hepatitis C virus infection, as well as those with cirrhosis and Child-Pugh class B liver function. Concerning tumor factors, all inflammation-based scores were significantly increased in patients with high serum AFP levels (≥200 ng/mL), a larger tumor (≥5 cm), the presence of vascular invasion, and an advanced tumor stage, but they were not significantly correlated with tumor number, except for SII.

During a median follow-up period of 20.5 months (IQR, 7.2–60.6 months), 583 patients (96.3%) experienced disease progression, and 424 patients (70.1%) died. The overall disease progression rate following TACE was 82.6% after 1 year, 91.8% after 2 years, and 94.6% after 3 years. The median TTP was 2.8 months (95% confidence interval [CI], 2.6–2.9). The overall cumulative death rate was 33.8% after 1 year, 49.4% after 2 years, and 57.9% after 3 years. The median OS was 24.4 months (95% CI, 19.2–29.6).

### 3.2. The Prognostic Values of the Inflammation-Based Scores and Fibrosis Markers after TACE

The prognostic values of the inflammation-based scores and fibrosis markers were analyzed. In the univariate analyses, increasing levels of all inflammation-based scores and fibrosis markers as continuous variables were significantly associated with a shorter TTP (all *p* < 0.05, Table 2). Among them, FIB-4 (HR, 1.02; 95% CI, 1.01–1.04; *p* = 0.008) and NLR (hazard ratio [HR], 1.06; 95% confidence interval [CI], 1.02–1.11; *p* = 0.007) were independent prognostic factors for disease progression in the multivariate analysis, along with AFP levels and tumor size and number, as well as the presence of vascular invasion. A time-dependent ROC analysis for predicting progression within 1 year showed that the cut-off values for FIB-4 and NLR were 3.0 and 1.7, respectively.

### 3.3. High NLR and FIB-4 Levels Predict a Shorter TTP after TACE

**Figure 1:**
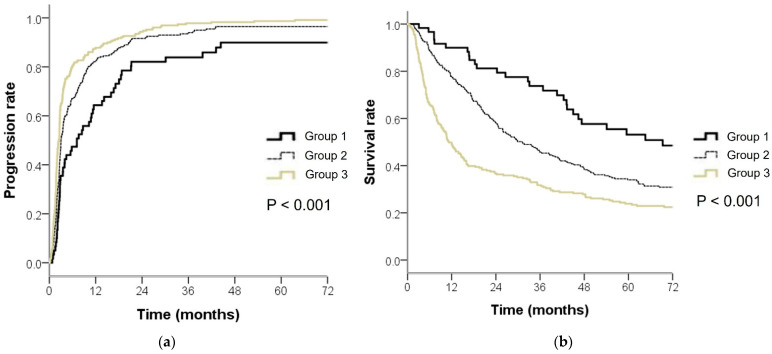
Kaplan–Meier estimates of (**a**) time to progression and (**b**) overall survival in patients with hepatocellular carcinoma after transarterial chemoembolization according to their neutrophil-to-lymphocyte ratio (NLR) and FIB-4 levels. Group 1, NLR < 1.7 and FIB-4 < 3.0; Group 2, NLR ≥ 1.7 and FIB-4 < 3.0 or NLR < 1.7 and FIB-4 ≥ 3.0; Group 3, NLR ≥ 1.7 and FIB-4 ≥ 3.0.

Because both inflammation and fibrosis are important perspectives in HCC, we evaluated the prognostic value of combining FIB-4 and NLR. Patients were divided into three groups as follows: group 1, patients with both low FIB-4 (<3.0) and NLR (<1.7); group 2, patients with either high FIB-4 (≥3.0) or high NLR (≥1.7); and group 3, patients with both high FIB-4 and NLR. The median TTP for groups 1, 2, and 3 was 7.1, 3.0, and 2.4 months, respectively, and 1-year progression rates for groups 1, 2, and 3 were 64.4%, 82.5%, and 91.6%, respectively (Figure 1a). As compared with group 1, group 2 had a 1.5-fold (HR, 1.53; 95% CI, 1.14–2.05; *p* = 0.004) increased risk of progression after TACE, and group 3 had a 2.1-fold (HR, 2.12; 95% CI, 1.58–2.86; *p* < 0.001) increased risk of progression after TACE.

### 3.4. High NLR and FIB-4 Levels Associated with Poor OS after TACE

The FIB-4 and NLR levels were also associated with patient survival. In the multivariate analysis, FIB-4 (HR, 1.02; 95% CI, 1.00–1.04; *p* = 0.02), NLR (HR, 1.09; 95% CI, 1.05–1.13; *p* < 0.001), Child-Pugh score, AFP levels, tumor size and number, and vascular invasion were independent prognostic factors for OS (Table 3). When the cut-off values for FIB-4 and NLR were applied to predict progression, the median OS for groups 1, 2, and 3 were 69.3, 31.2, and 11.3 months, respectively (Figure 1b). Group 2 had a 1.6-fold (HR, 1.62; 95% CI, 1.11–2.37; *p* = 0.01) increased risk of death, as compared with group 1, and group 3 had a 2.5-fold (HR, 2.58; 95% CI, 1.77–3.77; *p* < 0.001) increased risk of death, as compared with group 1.

## 4. Discussion

This study showed that higher baseline FIB-4 and NLR levels were associated with a shorter TTP, as well as decreased OS in patients with HCC who underwent TACE. These associations were independent of previously well-known prognostic factors such as the AFP level, tumor size and number, BCLC stage, vascular invasion, and Child-Pugh score.

In this study, the NLR, PLR, and SII levels gradually increased with a more advanced tumor stage and higher AFP levels, suggesting that increases in neutrophils and platelets or decreases in lymphocytes were associated with tumor progression. As inflammation-based scores are integrated based on circulating neutrophil, lymphocyte, and platelets counts, their prognostic values might be derived from the function of these cells. A recent study reported that neutrophils enhance tumor cell proliferation, angiogenesis, and metastasis, as well as their escape from immune surveillance [14]. Platelets have been known to facilitate tumor progression by inducing angiogenesis, sustaining cell proliferation, supporting cancer stem cells, and evading immune detection [15]. Meanwhile, relative lymphocytopenia might result in a poorer cell-mediated immune response to cancer [16]. Collectively, all these factors might promote tumor progression and escape from immune surveillance. Therefore, high levels of inflammation-based scores could reflect an HCC-promoting state, leading to a poor prognosis following TACE. Among the evaluated inflammation-based scores in this study, NLR was superior to PLR and SII and was an independent prognostic factor for both TTP and OS in patients with HCC who underwent TACE.

Several studies have reported the prognostic roles of noninvasive fibrosis markers after resection or RFA [7,17]. However, their impact on prognosis following TACE have rarely been evaluated. To our knowledge, there has been no study that evaluated the prognostic impact of FIB-4 in patients with HCC who underwent TACE. With regard to APRI, a recent study of patients with hepatitis C virus (HCV)-related HCC reported that a high APRI level (≥2.0) was independently associated with tumor recurrence, but not with survival after TACE [18]; however, another study involving mostly patients with HBV-related HCC demonstrated that the APRI level was associated with OS, but it was not significantly associated with disease-free survival [19]. Because these studies involved a relatively small number of patients (*n* = 274 and 191), a consistent prognostic impact has not been demonstrated regarding APRI. Compared with these studies, our study included a larger number of patients (*n* = 605) and showed that high FIB-4, rather than APRI, was an independent predictor of a shorter TTP and OS in patients with HCC who underwent TACE. Given that both chronic inflammation and fibrosis are important features shaping the tumor microenvironment, and they play important roles in the progression of HCC, combining the inflammation-based score with a fibrosis marker may enable more precise prognostication of patients than either use of a marker alone and be more useful for guiding the follow-up and subsequent treatment of patients with HCC who underwent TACE.

It is important to recognize TACE refractoriness/failure and provide patients with personalized therapeutics. For this reason, various models based on pre-treatment factors such as tumor characteristics, Child-Pugh score, AFP levels, and post-treatment parameters such as an increase in Child-Pugh score compared with baseline, radiologic tumor response, and the post-TACE transient hypertransaminasemia have been proposed as reliable prognostic tools [20,21,22,23,24,25]. These prognostic models, together with high baseline NLR and FIB-4 levels, might help individual prognostication and guide the decision process.

Our study had several limitations. First, as this was a retrospective study, we could not obtain data on other fibrosis markers and inflammatory cytokines. Combining other fibrosis markers with inflammatory cytokines may help in the more precise prediction of patient prognosis. Second, about one-third of patients had very early or early stage HCC. Thus, we may not have had a representative sampling of intermediate-to-advanced HCC, the standard target of TACE. However, this study aimed to assess the prognostic role of inflammation scores and fibrosis markers in all patients following TACE. Therefore, we did not exclude patients with very early or early stage HCC who were not candidates for resection or ablation and, thereby, underwent TACE. Third, the study was performed at a single institution; therefore, additional multicenter prospective studies are warranted for the generalizability of the results.

In conclusion, the present study showed that high baseline NLR and FIB-4 levels predicted the disease progression and death of patients with HCC who underwent TACE. The combination of these markers may help in the stratification of patients by capturing different perspectives on HCC, by focusing on fibrosis and inflammation.

## Figures and Tables

**Table 1:** Baseline characteristics of the study population.

Variables	Patients (*n* = 605)
Age (years)	57 (50–64)
Male	497 (82.1)
Cirrhosis	511 (84.5)
Etiology	
HBV	439 (72.6)
HCV	85 (14.0)
Non-viral	81 (13.4)
WBC (/mm^3^)	5200 (4065–6500)
Platelets (×10^3^/mm^3^)	127 (82–177)
Child-Pugh score	6 (5–7)
MELD-Na	10 (8–13)
Serum AFP (ng/mL)	110 (13–2150)
NLR	2.0 (1.4–3.1)
PLR	84.1 (63.1–125.2)
SII	248.3 (140.9–445.3)
APRI	1.3 (0.8–2.3)
FIB-4	4.4 (2.9–7.9)
Size of the largest tumor (cm)	3.7 (2.0–7.6)
Tumor number	
Single	276 (45.6)
Multiple	329 (54.4)
Vascular invasion	189 (31.2)
BCLC	
0	43 (7.1)
A	179 (29.6)
B	92 (15.2)
C	291 (48.1)

Data are median (interquartile range) or number (%), unless otherwise indicated. AFP, alpha-fetoprotein; APRI, aspartate aminotransferase-to-platelet ratio index; BCLC, Barcelona Clinic liver cancer; HBV, hepatitis B virus; HCV, hepatitis C virus; MELD-Na, model for end-stage liver disease-sodium; NLR, neutrophil-to-lymphocyte ratio; PLR, platelet-to-lymphocyte ratio; SII, systemic immune-inflammation index.

**Table 2:** Univariate and multivariate analyses of factors associated with progression.

Variables	Univariate Analysis		Multivariate Analysis	
	HR (95% CI)	*p*	HR (95% CI)	*p*
Age (≥60 vs. <60 years)	1.00 (0.99–1.01)	0.40		
Sex (male vs. female)	1.08 (0.89–1.34)	0.51		
Etiology		0.07		
HBV	0.87 (0.67–1.11)			
HCV	0.69 (0.50–0.95)			
Non-viral	1 (ref)			
Child-Pugh score	1.05 (0.98–1.11)	0.15		
AFP (≥200 vs. <200 ng/mL)	1.57 (1.32–1.86)	<0.001	1.23 (1.02–1.48)	0.03
Tumor size (≥5 vs. <5 cm)	2.45 (2.04–2.94)	<0.001	1.90 (1.53–2.36)	<0.001
Tumor number (≥ multiple vs. single)	1.73 (1.45–2.05)	<0.001	1.68 (1.41–2.02)	<0.001
Vascular invasion	2.48 (2.05–3.01)	<0.001	1.52 (1.20–1.93)	<0.001
BCLC stage		<0.001	-	0.20
0	1 (ref)			
A	1.34 (0.95–1.89)			
B	2.05 (1.41–2.98)			
C	3.04 (2.17–4.26)			
D	2.24 (1.33–3.78)			
NLR	1.12 (1.07–1.16)	<0.001	1.06 (1.02–1.11)	0.007
PLR	1.00 (1.00–1.00)	<0.001	-	0.86
SII	1.00 (1.00–1.00)	<0.001	-	0.27
APRI	1.03 (1.02–1.05)	<0.001	-	0.06
FIB-4	1.01 (1.00–1.03)	0.03	1.02 (1.01–1.04)	0.008

AFP, alpha-fetoprotein; APRI, aspartate aminotransferase-to-platelet ratio index; BCLC, Barcelona Clinic liver cancer; HBV, hepatitis B virus; HCV, hepatitis C virus; CI, confidence interval; HR, hazard ratio; MELD-Na, model for end-stage liver disease-sodium; NLR, neutrophil-to-lymphocyte ratio; PLR, platelet-to-lymphocyte ratio; SII, systemic immune-inflammation index.

**Table 3:** Univariate and multivariate analyses of factors associated with survival.

Variables	Univariate Analysis		Multivariate Analysis	
	HR (95% CI)	*p*	HR (95% CI)	*p*
Age (≥60 vs. <60 years)	1.00 (0.99–1.01)	0.46		
Sex (male vs. female)	1.19 (0.92–1.54)	0.19		
Etiology		0.39		
HBV	0.89 (0.67–1.17)			
HCV	0.78 (0.55–1.11)			
Non-viral	1 (ref)			
Child-Pugh score	1.32 (1.24–1.40)	<0.001	1.35 (1.26–1.45)	<0.001
AFP (≥200 vs. <200 ng/mL)	2.09 (1.73–2.54)	<0.001	1.45 (1.17–1.80)	0.001
Tumor size (≥5 vs. <5 cm)	3.31 (2.69–4.06)	<0.001	2.40 (1.87–3.08)	<0.001
Tumor number (≥multiple vs. single)	1.68 (1.38–2.04)	<0.001	1.53 (1.24–1.89)	<0.001
Vascular invasion	3.05 (2.50–3.72)	<0.001	1.86 (1.45–2.38)	<0.001
BCLC stage		<0.001	-	0.16
0	1 (ref)			
A	2.04 (1.19–3.50)			
B	3.43 (1.95–6.02)			
C	6.09 (3.60–10.31)			
D	10.05 (5.33–18.98)			
NLR	1.18 (1.15–1.22)	<0.001	1.09 (1.05–1.13)	<0.001
PRL	1.00 (1.00–1.00)	<0.001	-	0.25
SII	1.00 (1.00–1.00)	<0.001	-	0.08
APRI	1.03 (1.02–1.05)	<0.001	-	0.14
FIB-4	1.03 (1.01–1.04)	0.001	1.02 (1.00–1.04)	0.02

AFP, alpha-fetoprotein; APRI, aspartate aminotransferase-to-platelet ratio index; BCLC, Barcelona Clinic liver cancer; HBV, hepatitis B virus; HCV, hepatitis C virus; CI, confidence interval; HR, hazard ratio; MELD-Na, model for end-stage liver disease-sodium; NLR, neutrophil-to-lymphocyte ratio; PLR, platelet-to-lymphocyte ratio; SII, systemic immune-inflammation index.

## Data Availability

The data presented in this study are available on request from the corresponding author.

## Supplementary Materials

The following supporting information can be downloaded at: https://www.mdpi.com/article/10.3390/diagnostics12051170/s1, Table S1: The levels of inflammation-based scores and fibrosis markers according to clinical characteristics.
