# Supplementary material for: Prognostic Values of Inflammation-Based Scores and Fibrosis Markers in Patients with Hepatocellular Carcinoma Treated with Transarterial Chemoembolization
Source: Diagnostics (Basel). 2022 May 7;12(5):1170. doi: 10.3390/diagnostics12051170 (PMC9139803; doi:10.3390/diagnostics12051170)
Supplement: Supplementary file 1 [file diagnostics-12-01170-s001.zip › diagnostics-1675571-supplementary.pdf]

Supplementary Table S1. The levels of inflammation-based scores and fibrosis markers according to clinical characteristics

Variables	Patients (*n*=605)	NLR		PLR		SII		APRI		FIB-4	
		Median (IQR)	*P*	Median (IQR)	*P*	Median (IQR)	*P*	Median (IQR)	*P*	Median (IQR)	*P*
Age (years)			0.007		0.29		0.11		0.001		0.046
< 60	362	2.2 (1.5-3.3)		84.7 (64.3-129.2)		258.5 (145.1-456.6)		1.5 (0.8-2.7)		4.2 (2.5-8.0)	
≥ 60	243	1.9 (1.3-3.0)		82.8 (60.4-123.8)		236.0 (123.4-431.1)		1.1 (0.7-1.9)		4.7 (3.2-7.5)	
Gender			0.53		0.17		0.12		0.02		< 0.001
Male	497	2.1 (1.4-3.2)		82.9 (62.2-124.5)		250.7 (147.1-445.3)		1.3 (0.7-2.2)		4.2 (2.7-7.2)	
Female	108	2.1 (1.3-3.0)		94.6 (68.3-126.9)		209.2 (111.4-450.6)		1.6 (0.9-3.2)		6.3 (3.5-10.1)	
Etiology			0.001		0.001		< 0.001		< 0.001		0.001
HBV	439	2.1 (1.5-3.2)		84.4 (63.4-124.4)		250.7 (146.0-446.5)		1.3 (0.8-2.4)		4.4 (2.9-7.5)	
HCV	85	1.7 (1.0-2.4)		71.4 (53.0-102.9)		173.5 (98.1-295.0)		1.5 (0.9-3.1)		5.3 (3.7-9.5)	
Non-viral	80	2.1 (1.5-3.1)		105.7 (70.6-149.4)		368.1 (158.7-528.3)		0.9 (0.5-1.7)		3.4 (2.0-6.9)	
Cirrhosis[2]			0.38		< 0.001		< 0.001		< 0.001		< 0.001
Absent	94	2.1 (1.5-3.3)		105.8 (81.1-152.5)		371.2 (236.4-666.2)		0.9 (0.6-1.5)		3.2 (2.2-4.4)	

Present	511	2.1 (1.4-3.1)		81.4 (61.0-119.5)		224.4 (133.9-427.3)		1.4 (0.8-2.6)		5.0 (3.0-8.4)	
Child-Pugh class			< 0.001		0.42		0.63		< 0.001		< 0.001
A	463	1.9 (1.4-2.8)		84.8 (64.1-121.8)		247.9 (150.1-432.3)		1.1 (0.7-1.8)		3.7 (2.7-6.0)	
B	142	2.8 (1.7-4.1)		81.4 (55.2-131.6)		248.4 (102.8-508.9)		2.3 (1.4-4.2)		8.5 (5.0-12.6)	
Serum AFP (ng/mL)			< 0.001		< 0.001		< 0.001		0.97		0.06
< 200	342	1.9 (1.3-2.9)		77.9 (56.9-110.2)		199.0 (119.8-338.8)		1.3 (0.7-2.4)		4.8 (2.9-8.3)	
≥ 200	259	2.4 (1.6-3.6)		98.9 (74.4-145.9)		344.7 (199.7-612.7)		1.3 (0.8-2.2)		4.0 (2.8-7.2)	
Tumor size (cm)			< 0.001		< 0.001		< 0.001		0.52		0.003
< 5	355	1.8 (1.2-2.5)		74.7 (54.2-96.5)		187.1 (105.2-285.9)		1.3 (0.7-2.3)		4.8 (2.9-8.4)	
≥ 5	250	2.7 (1.8-4.0)		113.0 (79.6-157.7)		430.1 (232.0-710.6)		1.2 (0.7-2.2)		3.7 (2.6-6.5)	
Tumor number			0.22		0.07		0.004		0.57		0.91
Single	276	2.0 (1.3-3.3)		82.4 (60.1-118.5)		219.1 (118.8-411.4)		1.3 (0.7-2.2)		4.6 (2.7-8.0)	
Multiple	329	2.1 (1.5-3.1)		87.4 (64.7-132.4)		279.7 (156.9-468.7)		1.3 (0.8-2.5)		4.3 (3.0-7.5)	
Vascular invasion			< 0.001		< 0.001		< 0.001		0.25		0.39

Absent	416	1.9 (1.3-2.9)		77.9 (56.9-108.8)		206.1 (118.8-362.7)		1.3 (0.7-2.2)		4.7 (2.8-8.0)	
Present	190	2.7 (1.9-3.9)		111.2 (80.5-151.1)		412.6 (231.7-672.1)		1.4 (0.8-2.7)		4.1 (2.9-7.2)	
BCLC stage			< 0.001		< 0.001		< 0.001		0.04		0.004
0	43	1.7 (1.1-2.3)		68.3 (53.4-84.2)		167.2 (92.2-218.5)		1.3 (1.0-2.3)		5.3 (2.9-8.1)	
A	179	1.8 (1.2-2.5)		75.7 (54.6-99.7)		190.5 (103.7-298.2)		1.3 (0.8-2.2)		4.8 (3.0-8.4)	
B	92	1.9 (1.3-2.9)		79.4 (64.5-105.8)		250.1 (158.1-381.1)		1.1 (0.6-2.0)		3.4 (2.3-6.4)	
C	291	2.5 (1.8-4.0)		106.0 (74.2-150.1)		368.7 (185.4-648.0)		1.4 (0.8-2.5)		4.4 (3.0-7.4)	

AFP, α-fetoprotein; APRI, Aspartate aminotransferase-to-platelet ratio index; BCLC, Barcelona Clinic Liver Cancer; HBV, hepatitis B virus; HCV, hepatitis C virus; IQR, interquartile ranges; MELD-Na, Model for End-Stage Liver Disease sodium; NLR, neutrophil-to-lymphocyte ratio; PLR, platelet-to-lymphocyte ratio; SII, systemic immune-inflammation index.
